# Oxalate Content of the Herb Good-King-Henry, *Blitum Bonus-Henricus*

**DOI:** 10.3390/foods4020140

**Published:** 2015-05-12

**Authors:** Wanying Li, Geoffrey P. Savage

**Affiliations:** Food Group, Department of Wine, Food and Molecular Biosciences, Lincoln University, Lincoln 7647, Canterbury, New Zealand; E-Mail: Savage@lincoln.ac.nz

**Keywords:** total, soluble and insoluble oxalates, good-king-henry leaves, stems and buds, pesto, soup

## Abstract

The total, soluble and insoluble oxalate contents of the leaves, stems and buds of Good-King-Henry (*Blitum Bonus-Henricus*) were extracted and measured using HPLC chromatography. The large, mature leaves contained 42% more total oxalate than in the small leaves and the soluble oxalate content of the large leaves was 33% higher than the smaller leaves. Cooking the mixed leaves, stems and buds in boiling water for two minutes significantly (*p* < 0.05) reduced the total oxalate when compared to the raw plant parts. Pesto sauce made from mixed leaves contained 257 mg total oxalate/100 g fresh weight; this was largely made up of insoluble oxalates (85% of the total oxalate content). Soup made from mixed leaves contained lower levels of total oxalates (44.26 ± 0.49 mg total oxalate/100 g fresh weight) and insoluble oxalate made up 49% of the oxalate contents. The levels of oxalates in the Good-King-Henry leaves were high, suggesting that the leaves should be consumed occasionally as a delicacy because of their unique taste rather than as a significant part of the diet. However, the products made from Good-King-Henry leaves indicated that larger amounts could be consumed as the oxalate levels were reduced by dilution and processing.

## 1. Introduction

Good-King-Henry (*Blitum Bonus-Henricus* Syn. *Chenopodium Bonus-Henricus*) is a perennial herb in the *Chenopodium* family. It can be used as a food and a medicine; it is native to central and southern Europe. Other common names for the herb Good-King-Henry are perennial goosefoot, Lincolnshire spinach, markey, mercury, blite, wild spinach, early spinach, oak-leaved goosefoot, red goosefoot, common orache, long-stalked orache and spear-leaved orache. The edible parts of this plant are the young arrowed-shaped leaves, young flowering shoots and young flower buds. Since Good-King-Henry is in the same family as pig spinach (*Chenopodium* spp.), the leaves can be consumed in same way. However, it is better to consume the leaves in spring and early summer, as the old leaves become bitter and tough. The young and raw leaves can be chopped and mixed into salads. Leaves can also be cooked in the same way as spinach, by stir-frying, boiling and baking. Young flowering shoots can be cooked like asparagus and served in the same way. The flower buds can be prepared and cooked like broccoli; however, the buds are much smaller and it is tedious to harvest compared with broccoli.

The oxalate contents of Good-King-Henry leaves have not been measured previously but it would be reasonable to expect this plant would contain moderate to high oxalates as many other food plants in the *Chenopodium* family are known to contain these levels of oxalates in their leaves and stems. Goosefoot (*C. album*) has been reported to contain moderate to high levels of total oxalate levels in the raw leaves, ranging from 360 to 2000 mg/100 g dry matter (DM) [1], while Sood *et al.* [2] reported that the total oxalate contents of several different cultivars of goosefoot ranged from 394.2 to 518.4 mg/100 g DM. Beetroot (*Beta vulgaris* L. ssp.), *B. vulgaris* var. conditiva, mangold (*B. vulgaris* L. ssp. vulgaris var. vulgaris), spinach (*Spinacia oleracea*) and quinoa (*C. quinoa*) have all been shown to contain moderate to high levels of oxalates [3]. Siener *et al.* [3] show moderate oxalate levels in the edible parts of quinoa (*C. quinoa*) (184 mg/100 g total oxalate contents and 131 mg/100 g of soluble oxalate) and the root of beetroot (*B. vulgaris* L. ssp. vulgaris var. conditiva) contained 60 mg/100 g of total oxalate and 59.3 mg/100 g of soluble oxalate. In contrast, mangold (*B. vulgaris* L. ssp. vulgaris var. vulgaris) leaves, and spinach (*S. oleracea*) leaves, were shown to contain high levels of oxalates [3]. The mean total oxalate in mangold leaves was 874 mg/100 g fresh weight (FW) while the soluble oxalate was 327 mg/100 g FW. Spinach (*S. oleracea*) contained 1959 mg/100 g total oxalate and 1029 mg/100 g FW of soluble oxalate. Silver beet (*B. vulgaris* var. cicla) is also a widely consumed and popular leafy vegetable in the *Chenopodium* family. Savage *et al*. [4] showed that the mean total oxalate of three different raw coloured (white, red and yellow) leaves of silver beet was 792.7 ± 22.9 mg/100 g FW, which decreased to 659.9 ± 81.2 mg/100 g FW after boiling the raw leaves in tap water. There was no significant difference between the oxalate contents of the different coloured leaves. Finally, Noonan and Savage [5] reported that pig spinach (*Chenepodium* spp.) contained 1100 mg total oxalate/100 g FW and Siener *et al.* [3] reported that quinoa seeds (*C. quinoa*) contained 183–185 mg/100 g total oxalate. 

Oxalate is not an essential nutrient and is found in many kinds of edible plants with variable concentrations [5,6] and if consumed in large amounts, may be harmful to human health [5]. An intake of large amounts of soluble oxalate can increase the risk of kidney stone development because of the increased concentration of oxalate in the urine. As consumption of additional oxalate in the diet can cause the development of kidney stones in susceptible people it is important to identify high oxalate containing foods and, if possible, reduce these levels by processing [5]. In mammalian metabolism endogenous oxalate is produced by the breakdown of dehydroascorbic acid, glyoxylate, serine and glycine in the liver, and is excreted in the urine [7]. At moderate ascorbate intake levels, about 40% of the total oxalate excreted in the urine comes from the breakdown of ascorbate in the liver [7]. 

Savage *et al.* [6] showed that most high oxalate-containing foods contain both soluble (bound to Na^+^, K^+^ and NH_4_^+^) and insoluble (bound to Ca^2+^, Mg^2+^ and Fe^2+^) oxalates. Moreover, the soluble oxalates can bind to Ca^2+^, Mg^2+^ and Fe^2+^ ions to become insoluble salts during processing and cooking. Insoluble oxalates are excreted in the faeces while soluble oxalates are excreted via the kidneys.

The objective of this study was to investigate the distribution of oxalates in the leaves, as a function of leaf size, stems and buds of Good-King-Henry and to determine the oxalate content of the three different plant parts when cooked. The oxalate content of pesto and soup made from the leaves of the mature plants was also investigated.

## 2. Experimental Section

### 2.1. Harvesting and Cooking

Good-King-Henry seeds (*Blitum Bonus-Henricus*), were sown in plots early summer (November, 2013) in Wakanui silt loam in the Horticulture Research Area at Lincoln University, Canterbury, New Zealand (43°38′52.88″ S, 172°27′31.40″ E), 19 m above sea level. The soil was fertilised with chicken manure before sowing the seeds and the plots were irrigated as required throughout the growing period. In summer, early February 2014, mature plants (heights between 150 and 300 mm) were harvested. The plants were divided into three fractions, leaves, stems or buds and then divided further into two fractions: one fraction was immediately dried in an oven set at 105 °C for 24 h and the other set was boiled in tap water for 2 min, allowed to stand for 1 min, then drained and dried at 105 °C for 24 h. The leaves were cooked whole, while the stems were shopped into 10 mm segments. Both dried samples were ground to a powder using a Sunbeam multi grinder (Model no. EMO 400 Sunbeam Corporation Limited, NSW, Australia), and then sealed in plastic bags until analysis. Homemade pesto and soup were prepared by following the recipes in Table 1. All ingredients were placed in a blender and processed for 1 min. The soup mix was placed in a fry pan and simmered for 5 min with 250 mL of tap water until cooked. The homogenised pesto or the pesto soup were then frozen at −18 °C, and dried in a freeze dryer (W.G. Cuddon Ltd., Blenheim, Marlborough, New Zealand) and then finely ground in a coffee mill (Sunbeam, model EM 0400, China).

### 2.2. Moisture Content Analysis 

The moisture content analysis was carried out by following the AOAC method 935.10 [8].

**Table 1:** Ingredients for Good-King-Henry pesto and soup.

Spicy Good-King-Henry pesto	Good-King-Henry Soup
80 g leaves	46 g leaves
40 g garlic cloves	50 g chopped onions
40 g ground walnuts	6 g olive oil
8 g chilli sauce	28 g sour cream
60 g parmesan cheese	12 g ground rice
10 g olive oil	1 egg yolk
6 g salt	6 g salt
2 g pepper	250 mL tap water
150 mL tap water	

### 2.3. Oxalate Determination

The total and soluble oxalate content of 0.5 g of each finely ground sample was determined in triplicate using the method outlined by Savage *et al*. [6]. Oxalates were extracted from each raw and cooked sample of plant part. Soluble oxalate was extracted with 40 mL nanopure water (Barnstead II, Thermo Fisher Scientific Australia Pty Ltd., Scoresby, Victoria, Australia) and incubated in a water bath at 80 °C for 15 min, while total oxalates were extracted using 40 mL of 0.2 M HCl at 80 °C for 15 min. The extracted supernatants were filtered through a 0.45 mm cellulose nitrate filter. The chromatographic separation was carried out using a 300 × 7.8 mm Rezex ROA ion exclusion organic acid column (Phenomenex, Torrance, CA, USA) attached to a cation Hþ guard column (BioRad, Richmond, California, USA). The analytical column was held at 25 °C. The equipment consisted of an auto sampler (Hitachi AS-2000, Hitachi Ltd., Tokyo, Japan), a ternary Spectra-Physics, SP 8800 HPLC pump (Spectra-Physics, San Jose, CA, USA), a Waters, U6K injector (Waters Inc., Marlborough, MA, USA) and a UV/VIS detector Spectra-Physics SP8450 (Spectra-Physics, San Jose, CA, USA) set at 210 nm. Data capture and processing were carried out using a peak simple chromatography data system (SSI Scientific Systems Inc., State College, PA, USA). The mobile phase used was an aqueous solution of 25 mM sulphuric acid. Twenty microliters samples were injected onto the column and eluted at a flow rate of 0.6 mL/min. The insoluble oxalate content was calculated by difference [9]. The final oxalate values were converted to mg/100 g fresh weight (FW) of the original material, taking into account the moisture content of each sample. 

### 2.4. Standard Calibration 

Standard curves, containing 2–20 mg oxalic acid/100 mL in water and 0.2 M HCl were prepared and used to quantitate the soluble and total oxalic acid contents of the samples.

### 2.5. Statistical Analysis

All analyses were carried out in triplicate and the results are presented as mean values ± standard error. Statistical analysis of the oxalate data for the raw and cooked plant parts was performed using one-way analysis of variance (Minitab version 16, Minitab Ltd., Brandon Court, Progress Way, Coventry, UK).

## 3. Results and Discussion

The mean dry matter content of the large leaves and small leaves was 13.55% (Table 2). The Good-King-Henry samples contained high levels of dry matter in the leaves, stems and buds, and the values ranged from 15.5% to 16.3% (Table 3).

**Table 2:** The oxalate contents in raw large and small leaves (mg/100 g fresh weight (FW) ± standard error (SE)).Values in brackets are % soluble oxalate of total oxalate.

Plant part	Dry matter (%)	Total oxalate (mg/100 g FW)	Soluble oxalate (mg/100 g FW)	Insoluble oxalate (mg/100 g FW)
Large leaves	14.0	867.4 ± 15.8^a^	632.0 ± 27.6^a^ (72.9%)	253.4 ± 20.3
Small leaves	13.1	610.5 ± 5.3^b^	477.1 ± 25.9^b^ (78.1%)	157.1 ± 13.2

Values with a different superscript for the large and small leaves are significantly different (*p* < 0.05).

**Table 3:** The oxalate contents in raw and cooked leaves stems and buds of Good-King-Henry samples (mg/100 g FM ± SE). Values in brackets are % soluble oxalate of total oxalate.

Treatment	Plant part	% DM	Total oxalate (mg/100 g FW)	Soluble oxalate (mg/100 g FW)	Insoluble oxalate (mg/100 g FW)
Raw	Mixed leaves	15.5	703.5 ± 37.7 ^a^	465.7 ± 14.8 ^a^ (66.2%)	237.8 ± 23.4
	Stems	16.3	389.4 ± 28.0 ^a^	180.1 ± 7.2 (46.2%)	209.2 ± 21.5 ^a^
	Buds	15.9	332.8 ± 17.5 ^a^	264.1 ± 12.8 ^a^ (79.3%)	68.7 ± 26.0 ^a^
Cooked	Mixed leaves	10.1	281.9 ± 7.5 ^b^	91.3 ± 4.6 ^b^ (32.4%)	190.6 ± 12.0
	Stems	11.8	221.5 ± 1.5 ^b^	108.0 ± 27.1 (48.8%)	112.3 ± 26.6 ^b^
	Buds	14.1	233.1 ± 5.0 ^b^	29.8 ± 0.3^b^ (12.8%)	203.3 ± 4.8^b^

Data for the mixed leaves, stems and buds with a different superscript for the raw and cooked samples are significantly different (*p* < 0.05).

The total oxalate contents of the fresh leaves ranged from 610.5 ± 5.3 mg/100 g FW in the small leaves and from to 867.4 ± 15.8 mg/100 g FW in the large mature leaves, while the soluble oxalate ranged from 477.1 ± 25.9 mg/100 g FW in the small leaves and from to 632.0 ± 27.6 mg/100 g FW in the large leaves. The small leaves contained a higher proportion of soluble oxalate (78.1%), compared with 72.9% in the large mature leaves.

The mean value of total oxalate, soluble oxalate and insoluble oxalate contents of the raw and cooked samples of Good-King-Henry are presented on a fresh weight basis in Table 2. Both the raw and cooked mixed leaves contained higher levels of total oxalates when compared to the stems and buds. Overall, cooking reduced the total oxalate content when compared to the raw constituents. These values confirmed the observations made by Siener *et al.* [3], that plants in the *Chenopodium* family, accumulated higher levels of total and soluble oxalates in the leaves and stems compared with the roots and seeds.

After boiling in tap water for 2 min, the total and soluble oxalate contents in mixed leaves, stems and buds had significantly (*p* < 0.05) decreased, as the soluble oxalate was leached into the cooking water (Table 3). For example, there was 465.7 ± 14.8 mg/100 g FW (66.2% of total oxalate) soluble oxalate in the raw mixed leaves after cooking and the soluble oxalates were reduced to 91.3 ± 4.6 mg/100 g FW (32.4% out of total oxalate). There was also a significant (*p* < 0.05) decrease in the soluble oxalate contents of the raw buds, from 264.1 ± 12.8 mg/100 g FW to 29.8 ± 0.3 mg/100 g FW, in comparison to the cooked buds. In contrast, there was a large increase in insoluble oxalate content in the buds after cooking, a small increase in the stems and a fall in insoluble oxalates in the cooked mixed leaves. Similar observations have been made in earlier experiments where Savage *et al.* [4] analysed the oxalate contents of raw and cooked silver beet (*B. vulgaris* var. cicla). Good-King-Henry leaves can be used to make two different mixed products, pesto, which is not cooked, and soup, which is lightly cooked. While raw leaves of the Good-King-Henry samples were the main ingredient of the pesto and the soup, a range of other important ingredients were added (Table 1). The soup was then lightly cooked which reduced its moisture content marginally (the dry matters of the pesto and soup are 18.7% and 10.0%, respectively). The total oxalate content of the pesto was relatively high because 80 g of fresh leaves were mixed with other ingredients together with 150 mL of tap water. In contrast, the soup had much lower total oxalate content as it was made using 46 g of fresh leaves and other ingredients were added and then it was diluted with 250 mL water (Table 4).

**Table 4:** The oxalate contents in Good-King-Henry pesto sauce and soup (mg/100 g FW ± SE). Values in brackets % soluble oxalate of total oxalate.

Samples	Dry matter %	Total oxalate (mg/100 g FW)	Soluble oxalate (mg/100 g FW)	Insoluble oxalate (mg/100 g FW)
Pesto	18.7	257.1 ± 1.8	38.5 ± 0.4 (15.0%)	218.5 ± 2.0
Soup	10.0	44.26 ± 0.49	22.50 ± 0.29 (50.8%)	21.76 ± 0.35

The soluble oxalate content of the fresh raw leaves made up 66.2% of the total oxalate (Table 3). In contrast, the soluble oxalate content of the pesto was only 15% of the total oxalate (Table 4). This conversion of soluble oxalate during the preparation of the pesto to insoluble oxalate was initiated by the addition of 60 g Parmesan cheese to the recipe. 

The total oxalate content of the soup was relatively low because smaller amounts of fresh leaves (46 g) were added and the soup had 250 mL of tap water added. The proportion of soluble oxalate was reduced to 50% of the total oxalate, which was slightly lower than the 66.2% found in the fresh, raw leaves. This reduction in soluble oxalate occurred during the short cooking time (5 min) and was presumably initiated by the addition of 28 g of sour cream in the recipe, which contains a relatively lower amount of available calcium than Parmesan cheese, to initiate the conversion of soluble oxalate to insoluble oxalates.

The observation that Parmesan cheese, a high calcium containing food, can supply soluble calcium in the pesto sauce mix, which can then reduce the soluble oxalate content of the final mix, can be confirmed by earlier experiments where similar reductions in soluble oxalate occurred following the addition of milk and milk products. Mårtensson and Savage [10] conducted a study of the composition of oxalate in baked taro leaves cooked alone or with additions of cow’s milk or coconut milk. The results indicated that the total and soluble oxalate content of the baked leaves was significantly decreased after the leaves were baked with cow’s milk or coconut milk. They went on to report that a significant reduction of the soluble oxalate in baked leaves with milk occurred not only because of the dilution effects, but also from the binding of added calcium, which came from the cow or coconut milks, with the soluble oxalate from the baked leaves being formed into insoluble oxalate. 

Soup made from fresh Good-King-Henry leaves contained relatively low oxalate contents (Table 4). This was because few leaves were used (46 g) and more water was added to the mix (250 mL). The soluble oxalate was reduced more effectively in this mix as even the short cooking time may have allowed some soluble oxalate to be leached out of the leaves, but the conversion to insoluble oxalate by combining with soluble calcium released from the sour cream was less effective as it contained lower levels of calcium (100 mg Ca/100 g) compared with Parmesan cheese (1200 mg Ca/100 g) [11].

## 4. Conclusions

The total and soluble oxalate content of the large leaves of the herb Good-King-Henry were significantly (*p* < 0.05) higher than the smaller less mature leaves. As the herb, Good-King-Henry was a high oxalate-containing food it should only be consumed in small amounts especially by people who were prone to kidney-stone formation [12]. It was also advisable to prepare Good-King-Henry leaves with other calcium-rich foods to reduce the soluble oxalate content, thus, reducing the amount of oxalate absorbed into the body [13]. Pesto was an ideal way to prepare these leaves as the serving size was small, so low levels of oxalates will be ingested. Further work was required to determine the best method to prepare soup and pesto from these leaves to give a good flavour while maintaining a low soluble oxalate content. Informal tasting of the pesto and the soup made from the mixed leaves of Good-King-Henry had a pleasant sharp taste, which almost certainly came from the residual soluble oxalate in both of these products. 
